# A Case of an Immature PIT1-Lineage Pituitary Neuroendocrine Tumor of the Nasopharynx

**DOI:** 10.7759/cureus.44985

**Published:** 2023-09-10

**Authors:** Jessica Peters, Oscar Gryn, John Gerka-Stuyt

**Affiliations:** 1 Microbiology and Immunology, Des Moines University, Des Moines, USA; 2 Otolaryngology - Head and Neck Surgery, Western Reserve Hospital, Cuyahoga Falls, USA; 3 Otolaryngology - Head and Neck Surgery, University Hospitals Cleveland Medical Center, Cleveland, USA

**Keywords:** immature pit1 lineage pituitary neuroendocrine tumor, treatment of pituitary neuroendocrine tumor, somatostatin analog, nasal endoscopy with excisional biopsy, magnetic resonance imaging and pituitary neuroendocrine tumor, immunohistochemistry and biopsy, neuroendocrine tumor of the nasopharynx

## Abstract

Pituitary neuroendocrine tumors (PitNETs) located in the nasopharynx are a rare occurrence. This case report highlights a case of a 64-year-old female diagnosed with a PitNET incidentally found in her nasopharynx. The tumor was initially seen on MRI, excised, and analyzed with immunohistochemistry, ultimately confirming an immature pituitary-specific positive transcription factor 1 (PIT1)-positive-lineage pituitary neuroendocrine tumor. The tumor contained thyrotropes, somatotrophs, and lactotrophs expressing thyroid stimulating hormone, growth hormone, and prolactin, respectively. These tumors have the potential to exhibit aggressive behavior and can disrupt the surrounding tissue. Furthermore, they can be clinically silent or, conversely, secrete multiple hormones, causing hyperthyroidism, hyperprolactinemia, and acromegaly. For these reasons, they are deemed high risk. Treatment includes surgical excision with or without anti-hormone medications prior to surgery. Medications such as somatostatin analogs are used to decrease tumor size and reduce excessive hormone excretion.

## Introduction

Encountering pituitary neuroendocrine tumors (PitNETs) outside the sella turcica and in the nasopharynx is exceptionally rare [1]. The tumors are visualized on magnetic resonance imaging (MRI) with contrast. With histology and immunohistochemistry, PitNETs are diagnosed based on tumor lineage, morphological subtype, and whether they are clinically functional or nonfunctional. Plurihormonal pituitary-specific positive transcription factor 1 (PIT1) tumors co-secret in thyrotropes, somatotrophs, and lactotrophs. If they are clinically functional, symptoms can include acromegaly/gigantism, central hyperthyroidism, and hyperprolactinemia due to hormone hypersecretion. However, in most cases, these tumors are clinically silent [2-4].

Due to the silent nature of these tumors, patients rarely exhibit signs and symptoms of hormone hypersecretion. Early diagnosis of PitNETs is imperative as these tumors may be locally aggressive and destructive [2,3,5]. After establishing this diagnosis, many factors help guide treatment options. These factors include clinical signs of endocrine hypersecretion and neuroimaging findings, tumor immunohistochemistry, histology, and an assessment of cell proliferation. Treatment for these tumors involves resection and hormone-suppressing medications [2,3,6]. Ultimately, the prognosis of these tumors is questionable. These tumors are considered high risk as they can either be benign or aggressive and invasive [2].

## Case presentation

**Figure 1:**
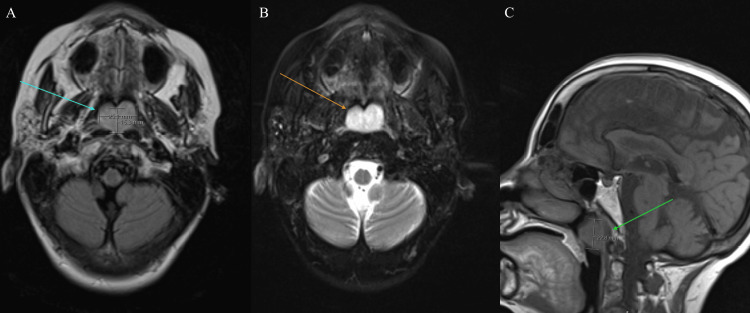
A prominent lobulated lesion measuring approximately 2.5 x 1.6 cm in cross-sectional dimension (A) within the nasopharynx, originating from the posterior pharyngeal wall, demonstrated hyperintense signals on FLAIR imaging and intense postcontrast enhancement (B). It measured 2.3 cm in craniocaudal dimension (C). FLAIR, fluid-attenuated inversion recovery

A 64-year-old female presented to the Ear, Nose and Throat department with a two-month history of left-sided tinnitus described as high-frequency buzzing that was intermittent and low in intensity. The patient also reported minor nasal congestion, posterior nasal drainage, and occasional shortness of breath. Her vital signs and subsequent basic metabolic panel were all within normal limits. Her past medical history was positive for tobacco abuse and hypertension, with medications including amlodipine, atorvastatin, escitalopram, losartan, and vitamin D3. She denied any past otolaryngologic history. The audiogram revealed a left normal sloping to moderate sensorineural hearing loss with high-frequency asymmetry. MRI of the internal auditory canal was ordered to evaluate for retrocochlear pathology, of which no positive findings were noted. However, an incidental nasopharyngeal mass was discovered (Figure 1). A nasal exam under anesthesia and an excisional biopsy of the nasopharyngeal mass were conducted. Flexible nasolaryngoscopy was performed with the scope inserted into the nasal cavity after adequate afrin and lidocaine spray. The nasal passage, nasal septum, turbinates, middle meatus, nasopharynx, and sinus ostia were visualized during the procedure. A large exophytic, homogeneous mass occupying most of the nasopharynx, which seemed pedunculated to the posterior septum, was noted and excised. No polyps or mucopurulent drainage was seen in the inferior meati, middle meati, or sphenoethmoidal recesses bilaterally.

Histologically, the tumor was periodic acid-Schiff stain negative and showed disrupted reticulin. After removing the tumor, patient labs showed an elevated chromogranin A level of 321 ng/mL (reference range, <39 ng/mL). Tumor immunohistochemistry exhibited positive focal growth hormone (GH) expression, positive variable prolactin (PRL) expression, and positive focal beta-thyroid stimulating hormone (beta-TSH) expression, as well as a PIT1-positive lineage (Table 1). An immature PIT1-lineage pituitary neuroendocrine tumor was diagnosed with infiltration of the nasopharyngeal mucosa. The lack of clinical symptoms and presumed lack of hormone secretion suggested a silent plurihormonal PIT1-positive pituitary neuroendocrine tumor [2].

**Table 1:** Tumor immunohistochemistry PIT1, pituitary-specific positive transcription factor 1; ER, estrogen receptor; GATA3, GATA-binding protein 3; SF1, steroidogenic factor 1; ACTH, adrenocorticotropic hormone; GH, growth hormone; PRL, prolactin; TSH, thyroid stimulating hormone; FSH, follicle stimulating hormone; LH, luteinizing hormone; TTF1, thyroid transcription factor 1; LI, labelling index

Protein	Result
PIT1	Positive
ER	Positive, variable
GATA3	Positive
SF1	Negative
ACTH	Negative
GH	Positive, focal
PRL	Positive, variable
Beta-TSH	Positive, very focal
Beta-FSH	Negative
Beta-LH	Negative
Keratin (CAM 5.2)	Variable
Keratin (AE1/3)	Negative
TTF1	Negative
Ki-67 LI	2.4%

**Figure 2:**
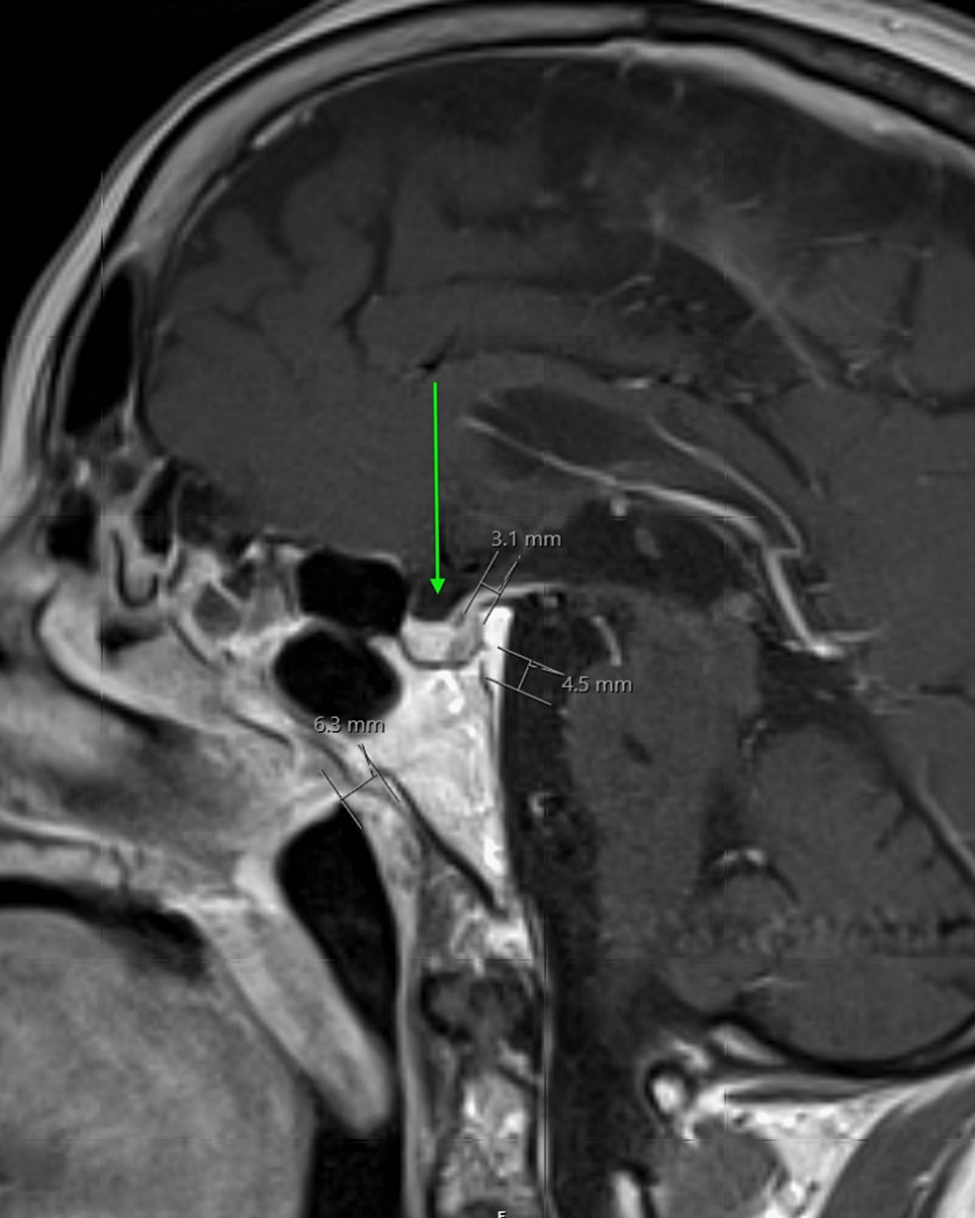
Post-operative MRI showing nonspecific hypoenhancing focus along the posterior aspect of the pituitary gland (arrow) and no hypointense signals in the posterior pharyngeal wall indicating complete excision of the tumor. Follow-up imaging could help distinguish the origin of the tumor, either an extension of the pituitary or ectopic.

The patient tolerated the procedure well with no complications. During the follow-up appointment, the patient reported resolution of nasal congestion and drainage, and her sense of smell improved. She had no changes in her hearing. Post-operative imaging showed complete tumor excision, but the tumor's origin could not be concluded as an extension of the pituitary or ectopic pituitary tissue (Figure 2).

## Discussion

A pituitary neuroendocrine tumor in the nasopharynx is an uncommon occurrence. Most of these tumors stem from the sella turcica [1]. Specifically, immature PIT1-lineage PitNETs express multiple hormones and can present symptomatically with tumor mass effect and hormone hypersecretion or, conversely, be asymptomatic. Diagnosing this tumor is difficult in cases where the patient is asymptomatic, and therefore, it is found incidentally. Symptomatic patients can present with hyperthyroidism, hyperprolactinemia, and acromegaly. Tumor mass effect refers to the disruption of surrounding anatomical structures and mucosa, which can manifest with mild symptoms such as posterior nasal drainage, like in our patient, or with a more severe form of mass effects such as visual disturbances [2,3].

MRI with contrast is used to evaluate a pituitary neuroendocrine tumor's invasion into surrounding structures and is the preferred initial diagnostic step. A subsequent biopsy is then performed to analyze tumor histology and evaluate the immunohistochemistry. Histologically, the tumor tissue is stained with hematoxylin and eosin (H&E), which allows for the visualization of the architecture, cellularity, and cytological features. It can distinguish between normal anterior and posterior pituitary tissues. Lastly, tumor immunohistochemistry evaluates tumor hormone expression, pituitary transcription factors, mitotic activity, and proliferation (Table 1) [2,3].

Tumor immunohistochemistry analysis will show a positive PIT1 tumor marker indicating a PIT1-lineage pituitary neuroendocrine tumor variably expressing GH, PRL, and beta-TSH (Table 1). In general, PIT1 is associated with estrogen receptors (ERs) in the differentiation of somatotroph, lactotroph, and thyrotroph cells during embryologic development [2]. A positive GATA3 biomarker is associated with a TSH-producing tumor, which supports a tumor's positive beta-TSH expression result [7]. Ki-67 LI is a nuclear protein used to determine the proliferation rate. A high proliferation rate indicates that the tumor is aggressive. A Ki-67 index ≥10% indicates an aggressive tumor and possible malignancy [2]. CAM 5.2 is a low-molecular-weight keratin. In one study analyzing the features of 31 silent plurihormonal PIT1 adenomas, previously called silent subtype 3 adenomas, CAM 5.2 was found in 90% of these tumors. In the study, all 31 of these tumors had disrupted the reticulin network and stained negative for periodic acid-Schiff, also seen in our patient's PitNET [7].

In our patient, MRI with contrast was used to visualize the tumor and its involvement within the posterior pharyngeal wall. Due to the lack of symptoms, no endocrine labs were drawn to assess for hormone hypersecretion before the tumor removal. Moreover, she showed no signs of hyperthyroidism, excessive prolactin levels, or increased growth hormone, all expressed in the tumor. Ultimately, it was unclear whether the tumor was producing hormones or was nonfunctional. Therefore, if this tumor is suspected, obtaining hormone levels should be considered in these patients as it helps classify the tumor and guide treatment options. Our patient's Ki-67 was 2.4%, indicating a low proliferative rate and a slow-growing tumor [2].

Two months after the tumor removal, our patient's lab values, including follicular stimulating hormone, luteinizing hormone, dehydroepiandrosterone sulfate, cortisol, thyroid stimulating hormone, free thyroxine, free triiodothyronine, prolactin, estradiol, calcitonin, insulin-like growth factor 1, thyroid stimulating immunoglobulin, antithyroid peroxidase antibodies and hemoglobin A1c, were all within normal ranges. In conjunction with the lack of initial symptoms and normal lab findings, this could indicate that the pituitary neuroendocrine tumor was nonfunctional and, therefore, silent. However, this could not be confirmed without initial labs before the tumor resection [2,3]. As far as the pituitary tumor's origin is concerned, more imaging must be conducted to determine if it is ectopic.

When evaluating a tumor in the nasopharynx, nasopharyngeal carcinomas should be considered. These masses present with symptoms related to mass effect that stem from their involvement in surrounding structures. These tumors are epithelial carcinomas arising from the mucosal lining of the nasopharynx. They are strongly associated with Epstein-Barr virus and other risk factors such as family history, tobacco smoking, high consumption of preserved foods, and poor oral hygiene [8].

Due to these tumors' infrequency and location, treatment protocols could be more precise. According to other case reports, the treatment for PIT1-positive, GH-, TSH-, and PRL-secreting tumors is initial control of hormone hypersecretion and surgical resection of the tumor. Specifically, in some case reports, treatment with somatostatin analogs like octreotide successfully reduced hormone secretion and inhibited tumor growth [4,6,9-12]. Because PitNETs can be large, invasive, and aggressive, preoperative somatostatin analog treatment can reduce the size of the tumor, making resection easier, and therefore reducing the need for further surgery [5]. In one case, carbimazole was used to treat hyperthyroidism before surgical tumor excision [13]. Dopamine receptor agonists like bromocriptine can be used in patients with hyperprolactinemia [14]. Lastly, radiotherapy is another option for resistant cases [11,12]. Surgery alone can be considered the treatment of choice. The patient discussed in this report was not experiencing signs and symptoms of hormone hypersecretion; therefore, tumor resection alone was sufficient for treatment, and no medication was indicated.

The prognosis depends on multiple factors. Imaging will indicate invasion into the surrounding structures and ascertain the size of the tumor. Because this tumor is atypical, being found in the nasopharynx, it makes the malignant potential uncertain. Tumor type, mitosis count, Ki-67 index, and p53 positive detection are also used in determining the prognosis. Silent plurihormonal PIT1-positive tumors are high risk as they can be aggressive. However, their malignancy risk remains unclear, and more evidence needs to be gathered on, specifically, how high risk these tumors are [2]. Treatment with surgery alone increases the risk of recurrence as these tumors can be large and invasive. Thus, treatment with a somatostatin analog can decrease the tumor size and improve successful removal. Finding these tumors early allows for the appropriate hormonal suppression therapy and surgical management to reduce the risk of metastatic disease [4,11].

## Conclusions

This case report highlights a rare case of an immature PIT1-lineage pituitary neuroendocrine tumor in the nasopharynx. The patient showed no coexisting clinical signs or symptoms of hormone hypersecretion, but a mild mass effect was noted. The tumor was found incidentally on MRI, excised, and analyzed with immunohistochemistry, ultimately confirming an immature PIT1-positive-lineage pituitary neuroendocrine tumor. General treatments include medications like somatostatin analogs to reduce tumor size and surgical excision. In this case, surgical management proved to be an excellent therapeutic option in treating the pituitary neuroendocrine tumor. Ultimately, this case report provides a unique perspective on neuroendocrine tumor presentations and treatment options.
